# Beauty and the beholder: the role of visual sensitivity in visual preference

**DOI:** 10.3389/fnhum.2015.00514

**Published:** 2015-09-23

**Authors:** Branka Spehar, Solomon Wong, Sarah van de Klundert, Jessie Lui, Colin W. G. Clifford, Richard P. Taylor

**Affiliations:** ^1^School of Psychology, UNSW AustraliaSydney, NSW, Australia; ^2^Department of Physics, University of OregonEugene, OR, USA

**Keywords:** fractals, fractal-like statistics, visual preference, aesthetics, 1/f amplitude spectrum, visual sensitivity, contrast sensitivity function

## Abstract

For centuries, the essence of aesthetic experience has remained one of the most intriguing mysteries for philosophers, artists, art historians and scientists alike. Recently, views emphasizing the link between aesthetics, perception and brain function have become increasingly prevalent (Ramachandran and Hirstein, 1999; Zeki, 1999; Livingstone, 2002; Ishizu and Zeki, 2013). The link between art and the fractal-like structure of natural images has also been highlighted (Spehar et al., 2003; Graham and Field, 2007; Graham and Redies, 2010). Motivated by these claims and our previous findings that humans display a consistent preference across various images with fractal-like statistics, here we explore the possibility that observers’ preference for visual patterns might be related to their sensitivity for such patterns. We measure sensitivity to simple visual patterns (sine-wave gratings varying in spatial frequency and random textures with varying scaling exponent) and find that they are highly correlated with visual preferences exhibited by the same observers. Although we do not attempt to offer a comprehensive neural model of aesthetic experience, we demonstrate a strong relationship between visual sensitivity and preference for simple visual patterns. Broadly speaking, our results support assertions that there is a close relationship between aesthetic experience and the sensory coding of natural stimuli.

## Introduction

Despite a long history of fascination with beauty and aesthetics, our understanding of many aspects of this experience remains, for the most part, elusive. Even widely agreed upon definitions of some of the associated terms are yet to emerge. For the purposes of the present research, the term aesthetics is not restricted to appreciation of artworks and is used more generally to refer to the attributes associated with visual appeal and preference of a wide range of natural and synthetic objects.

The work presented concerns the aesthetic appeal of images with fractal-like characteristics. Fractals feature patterns that repeat at increasing fine size scales. The term was introduced by Mandelbrot (1977) as an umbrella term encompassing exact fractals (which feature an exact repetition of patterns) and statistical fractals (for which only the statistical qualities repeat). Here we focus on the latter, which are prevalent in both mathematics and nature, including tree branches, coastlines, clouds, and mountain profiles. In contrast to the infinite magnification range for mathematical fractals, natural fractals repeat over a restricted range (Avnir, 1998; Mandelbrot, 1998). It is important to point out that there is no set minimum magnification range for determination of fractality *per se*. Instead, the required range is established by the repetitions sufficient to generate/observe the properties being investigated. For example, our previous studies showed that stable aesthetic properties could be observed for statistical fractals with magnification range as low as 25 (natural fractals) and considerably higher (Spehar et al., 2003; Taylor et al., 2011).

In addition to mathematical and natural fractals, there’s growing evidence that a number of artworks can be characterised as having fractal characteristics. Following the findings of Taylor and colleagues (Taylor et al., 1999) that Jackson Pollock’s poured paintings can be characterized by fractal-like scaling characteristics other researchers reported such properties in representational, abstract and graphic art (Mureika et al., 2004; Graham and Field, 2007; Redies et al., 2007). Graham and Field (2007, 2008) along with Redies et al. (2007) quantified the variation in intensity across the canvas using a Fourier spectrum analysis and found a 1/f^α^ dependence of the Fourier amplitude on the spatial frequency *f*. This power law reflects the image’s scale invariance and the exponent α quantifies its scaling properties. A wide range of art images and natural scenes were characterized by an intermediate α, with mean values of −1.23 and −1.40 respectively (Tolhurst et al., 1992; Graham and Field, 2007; Redies et al., 2007).

The underlying function of the fractal-like statistics found in artworks has received much attention. For example, Graham and Field (2008) proposed the “perceptibility hypothesis” in which the artist applies paint to maximize the visibility of the artwork’s structure, rather than its aesthetic appeal. A central proposition of the perceptibility hypothesis is that art images are rarely statistically random (i.e., quantified by *α* = 0) because random images are both difficult to perceive and to produce. Although images that show fractal-like 1/f scaling of natural scenes are just a small subset of all possible images, they are more prevalent because they “stand a better chance of being perceived” because our visual processing is matched to such spatial statistics. However, the perceptibility hypothesis “remains agnostic” as to the amount of aesthetic pleasure that is to be derived from images possessing particular fractal-like characteristics, and does not predict a preference for one fractal-like pattern over another (Graham and Field, 2008). In line with the perceptibility hypothesis, Mather (2014) has also proposed a central role for the artist’s visual system in adjusting the image spectral slope in artworks through either enhancing visibility or artistic rendering techniques which smooth out textural details while preserving edges.

Others have linked the fractal-like properties of images and artworks to their aesthetics (Spehar et al., 2003; Hagerhall et al., 2004; Redies, 2007). Referred to by Graham and Field (2008) as the “affect hypothesis, this second theory suggests that aesthetic perception and the efficient coding of sensory inputs might have the same underlying neural mechanisms: an aesthetically pleasing stimulus triggers a resonant state of neural activation (Redies, 2007).

Indeed, many studies have demonstrated a high degree of universality of aesthetic experience and its relation to the spatial properties found in natural scenes (Sprott, 1993; Aks and Sprott, 1996; Spehar et al., 2003; Hagerhall et al., 2004; Fernandez and Wilkins, 2008; Juricevic et al., 2010; Spehar and Taylor, 2013; Graham et al., 2015). Collectively, they show an aesthetic preference for intermediate values across a wide variety of images with fractal-like statistics. However, the mechanisms mediating preferences for spatial characteristics such as fractal scaling remain unclear. Preference for intermediate fractal scaling exponents is reminiscent of previous findings that patterns with moderate degrees of “complexity” are preferred (Fechner, 1876; Berlyne, 1971; Nadal, 2007; Forsythe et al., 2011). However, the extremely varied and sometimes imprecise definitions of spatial complexity in these studies make it challenging to integrate their findings, especially across different classes of images.

The aim of our study is to establish whether the preference for certain fractal-like statistics can be linked to the visual system’s general sensitivity for spatial variations across different scales. Our hypothesis is that early visual processing can mediate aesthetic judgments for different types of pattern and that sensitivity to image structure can be directly linked to aesthetic judgments. Specifically, we aim to show that the most preferred images contain structure with fractal-like statistics to which the human visual system is most sensitive.

Our proposal can be considered a variant of the well-known concept that aesthetic judgments are a function of the viewer’s processing fluency; that is, the quicker and more efficient the processing of the stimulus, the more positive the aesthetic judgment (Reber et al., 2004). This perspective considers aesthetic perception to be an interaction between an artwork’s objective properties and the observer’s processing characteristics of those properties. This view follows suggestions that “beauty is not “put in” the artwork as a distinct entity” (Zaidel, 2015), but rather it is “an emergent property in the brain of the beholder” where the beholder can include the artist as well as the observer (Redies, 2015). This view is also consistent with the neurophysiological approach (Ramachandran and Hirstein, 1999; Zeki, 1999, 2013; Livingstone, 2002; Ishizu and Zeki, 2013) suggesting that the mechanisms for aesthetic judgments are linked directly to functional characteristics of the visual brain.

We report three sets of experiments that investigate both visual sensitivity and visual preference for synthetic random images varying in their spatial scaling characteristics. By the direct comparison of these measurements using a within-subjects design, we can explore the potential relationship by which visual sensitivity may mediate visual preference. Experiment 1a compares visual preference and the visual system’s ability to detect spatial patterns varying in their amplitude spectrum characteristics. Experiment 1b examines the relationship between visual preference and the ability to discriminate between images varying in amplitude spectrum slope. Finally, Experiment 2 extends this hypothesis to the simplest visual patterns from the perspective of defining the visual system’s sensitivity for processing of spatial structure: sine wave gratings varying in spatial frequency. This latter experiment tests to what extent the relationship between visual sensitivity and visual preference is maintained for spatial variations that do not contain fractal structure or characteristics, but for which the visual system’s sensitivity has been well established.

## Experiment 1: Visual Preference and Visual Sensitivity for Synthetic Images Varying in Amplitude Spectrum Characteristics

As outlined above, the mechanisms mediating the visual preference for intermediate levels of fractal-like scaling are unclear. We propose a straightforward connection—that visual sensitivity mediates visual preference, and that this will be observable as heightened visual sensitivity to patterns that are visually preferred.

There have been a number of previous psychophysical attempts to determine the characteristics of the visual system’s processing of patterns with fractal-like statistics (Cutting and Garvin, 1987; Knill et al., 1990). Knill et al. (1990) measured human discrimination thresholds of 1/f^α^ amplitude spectrum patterns using a range of images with amplitude spectrum slopes, characterized by α, varying from 0.4–2.2. They found that changes in slope were most easily detected for images within the range 1.4 < α < 1.9, and observed decreasing performance on either side of this range. This inverse U-shaped function for discrimination of amplitude spectrum slopes, indicative of superior processing of images with intermediate fractal-scaling characteristics, has reappeared in several subsequent studies. Tadmor and Tolhurst (1994) found discrimination to be best for *α* = 0.8–1.0, and replicated this result in a later study (Tolhurst and Tadmor, 1997). Párraga et al., 2000, 2005), however, measured peak discrimination for α ~ 1.5, in agreement with the original study by Knill et al. (1990). It has been proposed that the variations in the exact range of amplitude spectrum characteristics associated with superior processing result from differences in the level of structure of the stimuli (Tadmor and Tolhurst, 1994), and projection of the stimuli to different portions of the retina that possess different sensitivities (Hansen and Hess, 2006).

In summary, it is apparent that the visual system’s ability to discriminate between 1/f amplitude spectrum patterns depends on the amplitude spectrum slope and that a similar dependence holds with respect to the visual preference for such patterns. However, in order to probe the suggested interrelationship more directly, we measure visual sensitivity and visual preference in the same observers and with the same stimulus patterns across sensitivity and preference judgments. We expect that this relationship should hold for different regimes of visual performance and we examine the relationship between visual preference and both absolute detection sensitivity (Experiment 1a) and discrimination sensitivity (Experiment 1b).

### Experiment 1a: Visual Preference and Absolute Detection Sensitivity for Synthetic Images Varying in Amplitude Spectrum

#### Method

##### Design

Experiment 1a employed a (2) (Visual preference, Detection sensitivity) × (6) (Amplitude spectrum slope variations) repeated measures design.

##### Apparatus

Stimuli were pre-drawn using a Visual Stimulus Generator (Cambridge Research Systems, Kent, UK) 2/5 graphics card, driven by MATLAB software. These were displayed on a 21 inch Sony Trinitron Multiscan (G520) monitor, operating at a frame rate of 100 Hz, with a resolution of 1280 × 1024 (20.1deg × 15deg). Display luminance was linearised using a 12-bit lookup table. A mean luminance of 58 cd/m^2^ was maintained throughout the duration of all trials in an otherwise dark environment. Viewing distance was fixed at 55 cm by placing the head in a chinrest. The stimuli in both tasks subtended a visual angle of 6 degrees. Responses were registered by pressing one of two buttons located on a response box.

##### Participants

Twenty-two undergraduate and postgraduate psychology students participated in the experiment in exchange for course credit. All participants were naïve to the purposes of the experiment and had normal or corrected-to-normal vision. The age of participants ranged between 17 and 30 years. All participants signed informed consent approved by the School of Psychology and UNSW Human Research Ethics Advisory Panels.

##### Stimuli

**Figure 1:**
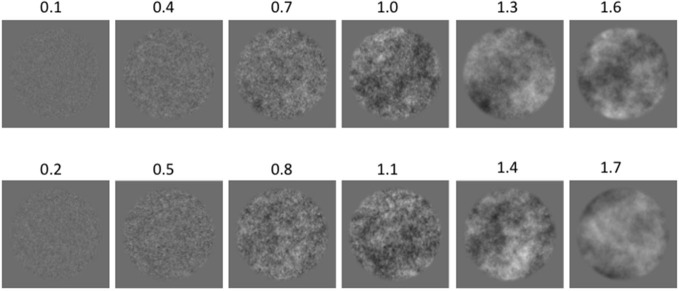
**Examples of 1/f amplitude-spectrum stimuli with increasing amplitude spectrum slopes (α): (top row) Series 1; (bottom row) Series 2**. All example images have the same mean luminance and the same Root Mean Square (RMS) contrast of 0.25 for the purpose of illustration of stimulus patterns.

The grayscale images were constructed by first creating a random noise pattern with each pixel value selected from a Gaussian distribution. A Fourier transform was then performed to obtain the amplitude frequency spectrum, which was adjusted to create a range of spectral slopes varying in increments of 0.3. Two series of experimental stimuli were created with Series 1 consisting of images with α values of 0.1, 0.4, 0.7, 1.0, 1.3, and 1.6, while Series 2 consisted of α values of 0.2, 0.5, 0.8, 1.1, 1.4, and 1.7. Half of the participants were tested with one α series and the remaining participants with the other, thereby obtaining a finer sampling of data points across the total range without exhaustively testing each participant. Examples from each amplitude spectrum slope α value are represented in Figure 1.

##### Absolute Detection Threshold Measurements

To determine the threshold contrast estimate at which each of the experimental images with varying amplitude spectrum slope was just detectable, we used the Psi procedure, a Bayesian adaptive method developed by Kontsevich and Tyler (1999). Each trial consisted of two consecutive intervals lasting 400 ms and on each trial observer performed a two-interval forced choice (2IFC) task. Determined randomly, one interval contained a cosine-ramped stimulus display while the remaining interval was empty and the observers were required to indicate via button-press which interval contained the stimulus. Two different auditory beeps marked the midpoints of the two intervals. Participants were given unlimited time to respond.

The trial-to-trial changes in stimulus contrast (expressed as root-mean-square, (RMS), contrast) were determined by the adaptive staircase procedure. This procedure estimates the observer’s contrast detection threshold as the level of 80% correct performance. Depending on the participant’s responses on previous trials, the stimulus contrast on each trial was chosen such that the participant’s response would be maximally informative in refining the threshold estimate. The number of trials to obtain threshold estimate for each amplitude spectrum slope value was set at 30 trials. For any given amplitude spectrum slope value α, threshold estimates were averaged across a minimum of three separate experimental runs. The detection thresholds were estimated for each of the six different amplitude spectrum slope values in separate blocks consisting of 30 trials, resulting in a total of 180 trials to generate threshold estimates for the six α values. The entire sequence was repeated at least three times with every observer, resulting in a minimum of 540 trials per each observer. Each repetition was conducted at least 30 min apart.

Before each experimental session, observers were familiarized with the task by performing one sequence of 30 practice trials.

##### Visual Preference Measurements

Visual preference was measured using the two alternative forced-choice (2AFC) paired comparison procedure. The paired comparison procedure was introduced by Cohn (1894), and is still considered a superior technique for measuring various forms of preference. Each trial consists of two images presented side-by-side and the task of the observers is to simply indicate (via key press) which of the two stimuli they visually prefer. The duration of the response interval was unlimited.

In this procedure, images of each amplitude spectrum slope α value are paired with all of the other five amplitude spectrum slope α values in its series, resulting in a total of 30 pairs. This basic sequence ensures that every experimental image is paired with every other and that each experimental image is presented 10 times across all experimental pairings with equal frequency on the left and the right side. All pairs were presented in random order and repeated three times, totaling 90 trials per experiment. A complete sequence of 90 trials was repeated at least twice for each observer, with some observers undergoing three or four repetitions each. For the visual preference measurements, all images were equated in their RMS contrast set at 0.30.

##### Procedure

Upon arrival, participants were provided with a general information sheet regarding the experiment, an informed consent forms, and written instructions for both of the experimental tasks. Participants completed the full experiment over periods ranging from 2–5 days.

#### Results and Discussion

For each image amplitude spectrum value, the visual sensitivity was expressed as an inverse detection threshold value and the visual preference was expressed as the proportion of 2AFC trials on which the image was chosen when presented.

Repeated measures one-way ANOVAs on the raw sensitivity and preference results revealed significant variations in both sensitivity and preference as a function of variations in the amplitude spectrum slope values in both data sets (Series 1 dataset: *F*_(5)_ = 15.92, *p* < 0.0001 and *F*_(5)_ = 8.599, *p* < 0.0046 for sensitivity and preference results, respectively; Series 2 dataset: *F*_(5)_ = 19.42, *p* < 0.0001 and *F*_(5)_ = 15.02, *p* < 0.0004 for sensitivity and preference results, respectively).

Both functions exhibit a typical inverted U-shaped characteristic, with a peak sensitivity and peak preference for the intermediate amplitude spectrum slope values. A posteriori Holm-Sidak multiple comparison *t*-tests were used to examine the pairwise differences in sensitivity and preference between different slope values. In Series A, detection sensitivity at the lowest (0.1) and the highest (1.6) slope values was statistically lower than for any other slope values (min *p* < 0.05; max *p* < 0.0001). In addition, the sensitivity at slope values of 0.7 and 1.0 was significantly higher than at slope value of 1.3 (*p* < 0.05). Likewise, in Series B, detection sensitivity at the lowest (0.2) and the highest (1.7) slope values were statistically lower than at values of 0.8 and 1.1 (min *p* < 0.05; max *p* < 0.0001). In addition, sensitivity at slope values of 0.8 and 1.1 was also significantly higher than at slope of 1.4. With respect to visual preference, in Series A the slopes of 1.0 and 1.3 were significantly higher than lower slope values of 0.1, 0.4 and 0.7. In Series B, the slope values of 1.1 and 1.4 were significantly more preferred than either lower slope values of 0.2 and 0.5 and the higher slope of 1.7 (min *p* < 0.05, max *p* < 0.0001). Taken together, with both stimulus sets we observe significantly higher sensitivity with intermediate than lowest and highest slope values. With respect to the visual preference with both stimulus sets we observe significantly higher preference with intermediate than lower slopes and significantly higher preference with slopes of 1.1 and 1.4 than the highest slope of 1.7 with the Series B. With the Series A, the highest slope value of 1.6 was not significantly less preferred than the intermediate slope values of 1.0 and 1.3. One possible reason for this is that the slope value of 1.6 is simply not “extreme” enough.

**Figure 2:**
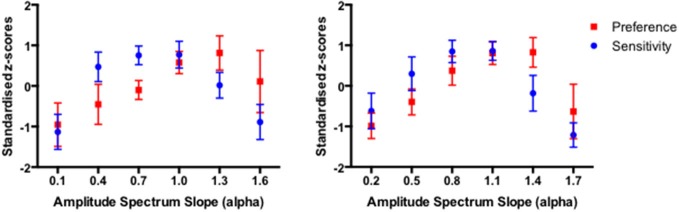
**The average sensitivity and preference results for 11 participants with Series 1 images (left panel) and 11 participants with Series 2 images (right panel)**. The average data points are expressed as the standardized *z*-scores with error bars representing 95% Confidence Intervals.

In order to compare the pattern of results from the two tasks on a common scale, the sensitivity (inverse detection threshold) and preference (proportion chosen) data were transformed into standardized *z* scores such that, for each individual observer, the corresponding sensitivity and preference data sum to 0 across the amplitude spectrum slope values. The panels in Figure 2 show the average sensitivity and preference standardized scores for 11 participants with Series A images (left panel), and 11 participants with Series B images (right panel). The error bars correspond to 95% Confidence Intervals associated with the respective condition means. For both data sets, a (2) × (6) repeated measures ANOVA revealed significant main effect of slope (*F*_(5)_ = 21.25, *p* < 0.0001 and *F*_(5)_ = 25.45, *p* < 0.0001 for Series 1 and Series 2 data sets respectively). Given that the raw scores in the detection sensitivity and visual preference were standardized, the comparison between the task is not meaningful (with the *F* ratio of 0 in both data sets) However, for both data sets there was a significant Slope × Task interaction (*F*_(5,50)_ = 6.407, *p* < 0.0001 and *F*_(5,50)_ = 7.535, *p* < 0.0001 for Series 1 and Series 2 data sets respectively).

While the overlap between the average sensitivity and preference results in each data set is considerable, the significant Slope x Task interaction indicates that the peaks of the sensitivity and preference functions differ somewhat. Nevertheless, the correlation between the average sensitivity and preference for the combined amplitude spectrum slope values across Series 1 and Series 2 (12 pairs in total) reflects a moderate association with Pearson correlation coefficient r equaling 0.55 (*p* < 0.064). The correlation coefficients for the two data sets separately equal 0.43 and 0.66 for Series 1 and Series 2 data sets respectively, but do not reach significance due to a very small number of pairs (6) in each case.

**Figure 3:**
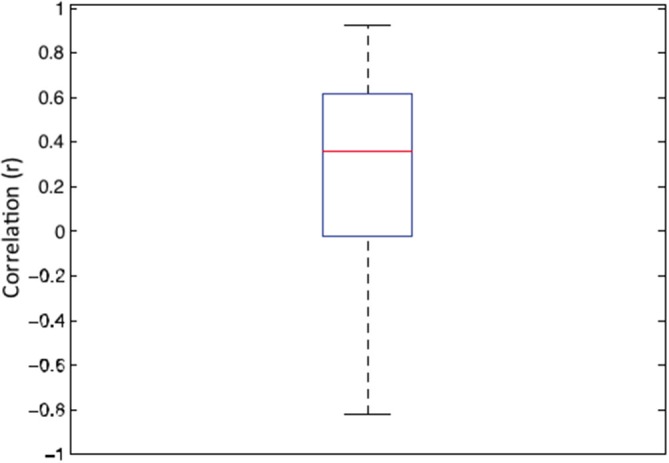
**Box and whisker plot of correlation coefficients between sensitivity and preference data for 22 individual observers**. In this plot, the 25–75 percent quartiles of the distribution are drawn using a box. The median is shown with a horizontal line inside the box. Maximum “whisker” length corresponds to 1.5 *Interquartile range (25–75). This value corresponds approximately to ± 2.7 SD and 99.3% coverage. The points outside of this range are considered outliers.

It has been argued that, in general, correlated averages are not informative about the association between the two variables at the level of each individual observer (Thorndike, 1917; McManus, 1980; McManus et al., 2010; Vessel and Rubin, 2010). Therefore, in order to establish whether the association sensitivity and preference exist beyond aggregation, we first calculated the individual correlation coefficients for each of 22 observers. The average of these individual correlations between sensitivity and preference was 0.342 (95% *CI* = 0.123–0.525; *t*_(21)_ = 3.351, *p* < 0.003). Figure 3 depicts the box and whiskers plot of individual correlations between detection sensitivity and preference for 22 observers. The median of individual correlations between detection sensitivity and visual preference was 0.358.

In summary, the peak detection sensitivity and visual preference both occurred for intermediate amplitude spectrum slope values but did not overlap completely. Overall, we found modest but significant association between sensitivity and preference for synthetic images varying in their amplitude spectra at both group and individual levels. It is quite remarkable that we were able to observe these significant levels of correlation given the very different nature of stimulus presentation: 400 ms in the absolute detection sensitivity task vs. unlimited exposure in the visual preference task. The experience with the stimuli was also quite dramatically different in the two task conditions, given the near invisibility of patterns presented at detection threshold vs. the high contrasts used in the visual preference task.

### Experiment 1b: Visual Preference and Discrimination Sensitivity for Synthetic Images Varying in Amplitude Spectrum

In Experiment 1b we test the generality of the sensitivity-visual preference association with a different measure of visual sensitivity and across a wider range of variations in the slopes of the amplitude spectra. Here we examine the ability to discriminate differences in the amplitude spectrum slope of synthetic images, now presented at high supra-threshold contrast and hence clearly visible. We measure the size of just noticeable differences (JNDs) necessary to perceive increases and decreases in the amplitude spectrum slope at reference values ranging from 0.5–2.5 (with incremental steps of 0.25). As in the previous experiment, visual preference was measured for the same range of amplitude spectrum exponent values.

#### Method

##### Design

As in the previous experiment, Experiment 1b employed a (2) (Visual preference, Detection sensitivity) × (6) (Amplitude spectrum slope variations) repeated measures design.

##### Apparatus

Testing was done using a HPZ230 workstation with Intel Core i7 processor, connected to a 24-inch LED Benq monitor, set at its native resolution of 1920 × 1080, at 100 Hz. The experimental stimuli and procedures were created using Matlab software with the Psychophysics Toolbox extensions (Brainard, 1997). Participants were seated at a viewing distance of 55 cm with the viewing position stabilized using a chinrest.

##### Participants

Forty-six undergraduate psychology students participated in the experiment in exchange for course credit (16 males and 30 females). None of the participants took part in the previous experiment and all participants were naïve to the purposes of the experiment and had normal or corrected-to-normal vision. The age of participants ranged between 17 and 30 years. All participants signed informed consent approved by the School of Psychology and UNSW Human Research Ethics Advisory Panels.

##### Stimuli

**Figure 4:**
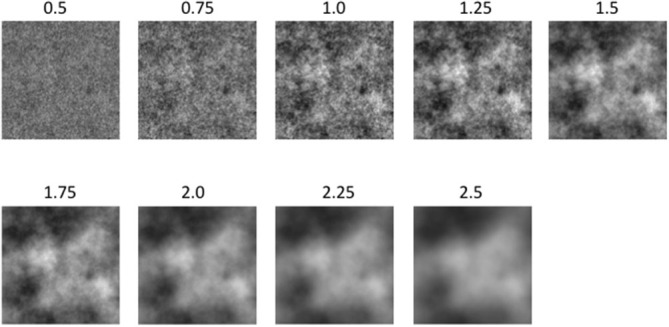
**A subset of the experimental image used in Experiment 1b: From left to right the amplitude spectrum slope values are: 0.5, 0.75, 1.0, 1.25, 1.5, 1.75, 2.0 and 2.5**.

The grayscale images were constructed by the same procedure as outlined in Experiment 1a, with α values of 0.5, 0.75, 1.0, 1.25, 1.5, 1.75, 2.0, 2.25 and 2.5. For each amplitude spectrum slope value α, we generated three different versions or exemplars which were randomized across observers. One complete series of grayscale images is depicted in Figure 4.

##### Discrimination Threshold Measurements

To determine the discrimination threshold contrast (JND) necessary to be able to detect increases and decreases in the amplitude spectrum slope of the reference image, we again used the Bayesian adaptive Psi procedure (Kontsevic and Tyler, 1999). For the purpose of determining JND thresholds, we employed a four Alternative Forced Choice, “odd-one-out” task, in which the observer was asked to find the stimulus which was different among four images shown on any given trial.

**Figure 5:**
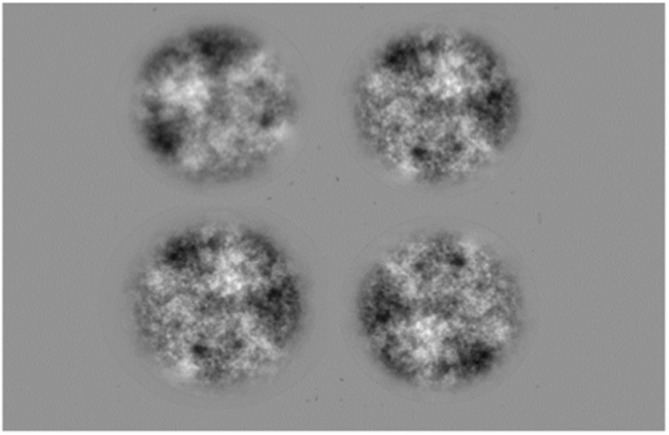
**Example of an experimental trial: Three quadrants have filtered noise patterns with an amplitude spectrum slope of −1.25 and one quadrant (top left) has a filtered noise pattern with an amplitude spectrum slope of −1.5**.

Each individual trial began with a fixation point at the centre of the screen for 500 ms. The fixation screen was followed by a trial display in which the four images were shown for a period of 500 ms. Each image was presented in a circular aperture with a blurred (raised cosine) edge as illustrated in Figure 5. In each trial, three of the images had the baseline amplitude spectrum slope which remained the same throughout a block. The remaining, odd-one-out, image had a different amplitude spectrum slope, determined according to the participants’ previous responses, and it appaeard randomly with equal probability in each of the four quadrants. All of the stimuli were rotated relative to one another instead of repeating the same pattern with the same orientation. This was done to encourage subjects to focus on the overall appearance of the stimulus rather than to adopt an image matching strategy on local regions of the stimulus. The duration of the response interval was unlimited.

This phase contained 9 blocks, and within each block, the discrimination threshold was determined for one of the reference amplitude spectra slopes. The blocks with different reference slopes were run in a randomized order.

##### Visual Preference Measurements

Visual preferences were determined with a paired comparison procedure as described in Experiment 1a. All images were equated in RMS contrast set at 0.30. Each of the images with different amplitude slope value was paired with all other eight images from resulting in 72 pairs for each image class. Within the 72 trials, each image was presented equally often on the left and the right side and was presented a total of 16 times. The participants indicated which image in the pair they preferred by a button press.

#### Results and Discussion

As in the previous experiment, visual sensitivity was expressed as an inverse detection threshold value and the visual preference was expressed as the proportion of paired comparison trials on which the image was chosen when presented.

First, one-way repeated measures ANOVAs were performed with the raw discrimination sensitivity and visual preference data. Both the discrimination thresholds and the visual preference for grayscale images varied significantly as the function of the amplitude spectrum slope (*F*_(8)_ = 147.1, *p* < 0.0001 and *F*_(8)_ = 9.264, *p* < 0.0002 for visual discrimination and visual preference respectively).

As in Experiment 1a, we performed a posteriori Holm-Sidak multiple comparison *t*-tests in order to confirm the inverted U-shaped characteristic for both discrimination sensitivity and the visual preference functions. For the data regarding the discrimination sensitivity, out of 36 pairwise comparisons all but four were statistically significant (min *p* < 0.05; max *p* < 0.0001). Non-significant differences were observed between the extreme slope values of 0.75 and 2.5 as well as between slope values of 1.25 and 2.25; 1.5 and 2.25; and 1.75 and 2.0. Taken together, these comparisons confirm the inverted U-shape function of discrimination sensitivity as a function of amplitude spectrum of 1/f grayscale images. With respect to the visual preference for the Grayscale images, the two intermediate slope values of 1.25 and 1.5 were significantly more preferred compared to the two lowest (0.5 and 0.75) slope values and the two highest (2.25 and 2.5) slopes values. The slope value of 1.75 was also significantly more preferred than the lowest slope value of 0.5 and the higher slope values of 2.0, 2.25 and 2.5, thus adding support for the inverted U-shaped distribution of visual preferences for the Grayscale images.

**Figure 6:**
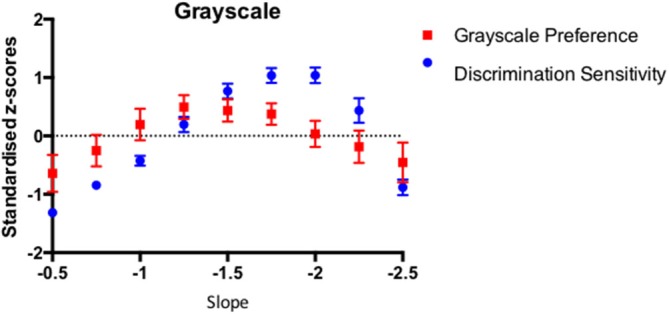
**The average discrimination sensitivity and visual preference results for 46 participants plotted as a function of the amplitude spectrum slope of the Grayscale images**. The average data points are expressed as the standardized *z*-scores with error bars representing 95% Confidence Intervals.

In order to compare the pattern of results from the sensitivity and preference tasks on a common scale, the sensitivity (inverse detection threshold) and preference (proportion chosen) data were transformed into standardized *z* scores such that, for each individual observer, the corresponding sensitivity and preference data sum to 0 across the amplitude spectrum slope values. Figure 6 shows the average standardized scores for sensitivity and preference for 46 participants with the Grayscale images. The error bars correspond to 95% Confidence Intervals associated with the respective condition means.

We performed a (2) Task x (9) Exponent repeated measures ANOVA to test whether the sensitivity and preference scores vary in the same way as a function of the image amplitude spectrum exponent. The analysis revealed significant main effect of slope (*F*_(8)_ = 37.49, *p* < 0.0001). As discussed before, given that the raw scores in the detection sensitivity and visual preference were standardized, the comparison between the tasks is not meaningful. As in Experiment 1a, the Slope x Task interaction was significant (*F*_(8,360)_ = 10.62, *p* < 0.000).

**Figure 7:**
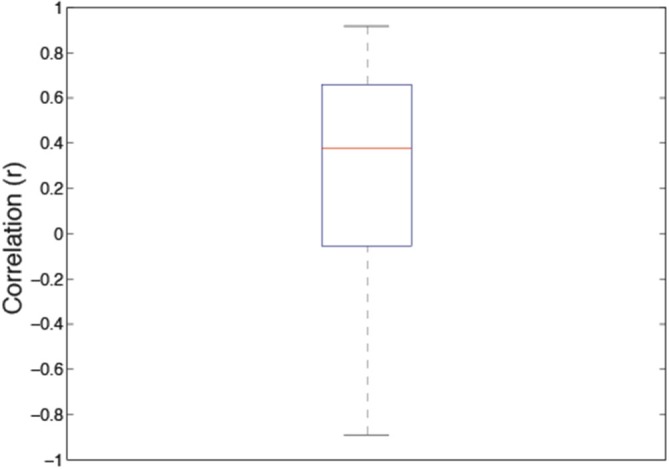
**Box and whisker plots of individual correlations between discrimination sensitivity and preference for 46 observers**. The 25–75% quartiles of the distribution are drawn using a box. The median is shown with a horizontal line inside the box. Maximum “whisker” length corresponds to 1.5 *Interquartile range (25–75). This value corresponds approximately to ± 2.7 SD and 99.3% coverage. The points outside of this range are considered outliers.

The correlation between the average discrimination sensitivity and visual preference for the Grayscale images was quite high with Pearson *r* equaling 0.738 (*p* < 0.023). However, in order to establish whether the association sensitivity and preference exist beyond level of average results, we calculated the average of individual correlation coefficients across 46 observers. The average individual correlations between discrimination sensitivity and preference for 46 participants equaled 0.267 (95% *CI* = 0.127–0.406, *p* < 0.0004). Figure 7 depicts the box and whiskers plot of individual correlation coefficients between discrimination sensitivity and preference data for 46 individual observers. As indicated in this figure, the median of individual correlations between discrimination sensitivity and visual preference was 0.378.

As we observed before, the peak detection sensitivity and visual preference both occurred for intermediate amplitude spectrum slope values and we found significant association between sensitivity and preference for synthetic images varying in their amplitude spectra at both group and individual levels.

## Experiment 2: Visual Preference and Visual Sensitivity for Sine-wave Gratings Varying in Spatial Frequency

**Figure 8:**
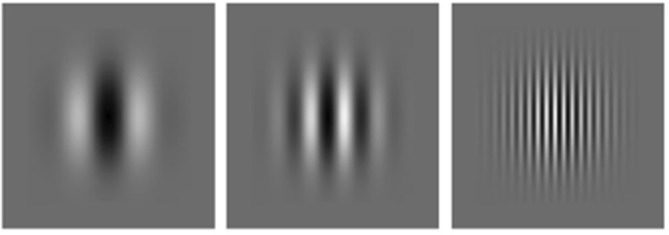
**Sine-wave gratings varying in spatial frequency from left to right**.

While our hypothesis that visual sensitivity mediates visual preference ties in well with the recent interest and findings around natural image statistics, we propose that it also applies to other forms of image structure. To directly test the hypothesis that visual preference varies as a function of spatial variations for which the visual system exhibits differential sensitivity we turn to the simple sine-wave configurations ranging from low to intermediate and high spatial frequency depicted in Figure 8 as the most suitable configurations.

The contrast sensitivity function that maps the normal adult human visual system’s sensitivity to sine-wave grating varying in spatial frequency is well-documented. The function is characterised by an inverse U-shaped function, with a sharp drop in sensitivity at higher spatial frequencies and a gentler drop at lower spatial frequencies. While the population average is widely considered to peak at or near 4 cycles per viewing degree (c/d), individual peaks vary between 2 and 6 c/d (Campbell and Robson, 1968; Graham, 1989; De Valois and De Valois, 1990; Field and Brady, 1997). The shape and peak of the contrast sensitivity function are relatively stable but can, however, be affected by changes such as location of retinal presentation and luminance level. Interestingly, Bex et al. (2009) have reported that contrast sensitivity is quite different when measured against natural images as a background, with a pronounced selective loss at low spatial frequencies.

In order to be able to establish a direct measure of the strength of co-variation between visual sensitivity and visual preference for simple sine-wave gratings, we again measure both sensitivity and preference in the same set of observers and for the same patterns.

### Method

#### Design

As in the previous two experiments, a (2) (Visual preference, Detection sensitivity) × (7) (Spatial frequency) repeated measures design was employed.

#### Participants

Twenty-nine first year psychology students participated in the experiment in exchange for course credit. Twelve of these participants also received financial compensation. All participants were naïve to the purposes of the experiment and had normal or corrected-to-normal vision. The age of participants ranged between 17 and 44 years. Informed consent approved by the UNSW Human Research Ethics Advisory Panel was obtained.

#### Stimuli

All stimuli were constructed using MATLAB (version 6.12) and corresponding Psychophysics Toolbox (version 2.5). Stimuli consisted of Gaussian enveloped sine-wave gratings with peak spatial frequency ranging from 0.25, increasing in multiples of two, to 16 cycles per viewing degree when presented at 54 cm viewing distance. Phase was randomised with each presentation in both the contrast detection and visual preference tasks.

#### Absolute Detection Threshold Measurements

To determine the threshold contrast at which each of the sine-wave gratings with varying spatial frequency was just detectable, we used the same Psi procedure as described in Experiment 1a. The average thresholds for the whole stimulus set were based on at least 4 (and up to 6) runs with every observer.

#### Visual Preference Measurements

As before, the visual preference was measured using the 2AFC paired comparison procedure. In order to control for the possibility that participants would choose a sine-wave grating of middle spatial frequency as preferred even in the absence of any strong visual preference we introduced a modification to ensure that the visual preference results are not affected by range effects or the “regression to the mean” strategy. Namely, the testing blocks for the visual preference task did not use the entire range of spatial frequency manipulations, and instead we created two sequences that span either low to mid spatial frequency range (0.25, 0.5, 1, 2 and 4 c/deg) or mid to high spatial frequency range (1, 2, 4, 8 and 16 c/deg). Each of the two sequences consisted of 20 unique pairs, repeated three times in random order. Each participant completed both sequences at least twice.

### Results and Discussion

**Figure 9:**
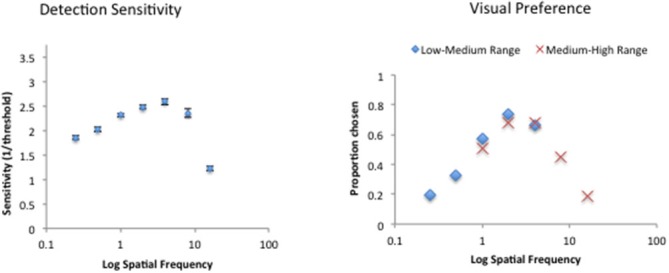
**Raw detection sensitivity (left) and visual preference for sine-wave gratings varying in spatial frequency for 29 participants**.

Figure 9 depicts the average sine-wave detection sensitivity and visual preference data. The panel on the left shows the average inverse contrast detection thresholds as a function of spatial frequency with the characteristics of a typical contrast sensitivity function: an inverse U-shaped function with a peak in the intermediate range, in this case at 4c/deg. The panel on the right shows the average visual preference results for the stimuli in two experimental sequences and shows that the results were not influenced by the range effect or the tendency to rate the spatial frequencies in the middle of each sequence as the most preferred. Instead, the visual preference for the range of spatial frequencies present in both experimental sequences was equal regardless of the experimental sequence they belonged to. For the subsequent data analyses, the visual preference results from the two sequences were combined.

Repeated measures ANOVAs were performed with the raw detection sensitivity and visual preference data as a function of spatial frequency. The detection sensitivity varied significantly as a function of the amplitude spectrum slope (*F*_(6)_ = 188.7, *p* < 0.0001). Similarly, the visual preference for sine-wave gratings also varied significantly as a function of spatial frequency (*F*_(6)_ = 83.95, *p* < 0.0001). A posteriori Holm-Sidak multiple *t*-tests revealed that detection sensitivity was significantly higher with the intermediate spatial frequencies of 2 and 4 cycles per degree compared with the two lowest (0.25 and 0.5cpd) and the two highest (8 and 16 cpd) spatial frequencies. Similarly, intermediate spatial frequency of 4 cpd was significantly more preferred than the two lowest (0.25 and 0.5 cpd) and the two highest (8 and 16 cpd) spatial frequencies.

**Figure 10:**
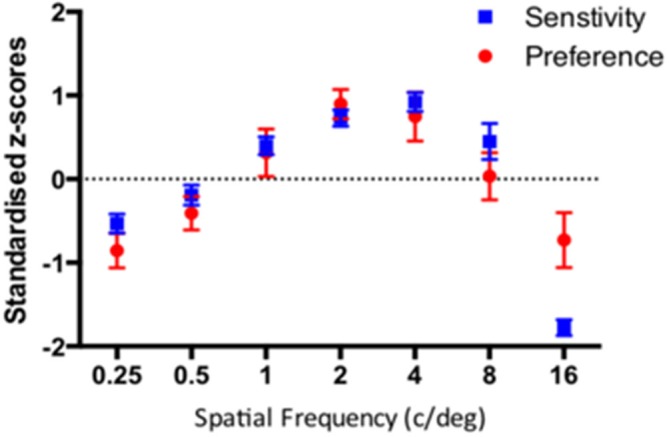
**Standardised sensitivity and visual preference for sine-wave gratings varying in spatial frequency for 29 participants**. The error bars correspond to 95% Confidence Intervals associated with their respective condition means.

As in the previous experiments, we compare the pattern of results from the sensitivity and preference tasks via the standardized *z* scores as shown in Figure 10. Both functions exhibit remarkably similar characteristics with peaks around 2–4c/deg.

A (2) Task x (7) Spatial Frequency repeated measures ANOVA revealed significant main effect of spatial frequency (*F*_(6)_ = 100.9, *p* < 0.0001) and a significant spatial frequency by task interaction (*F*_(6,168)_ = 11.36, *p* < 0.0001).

**Figure 11:**
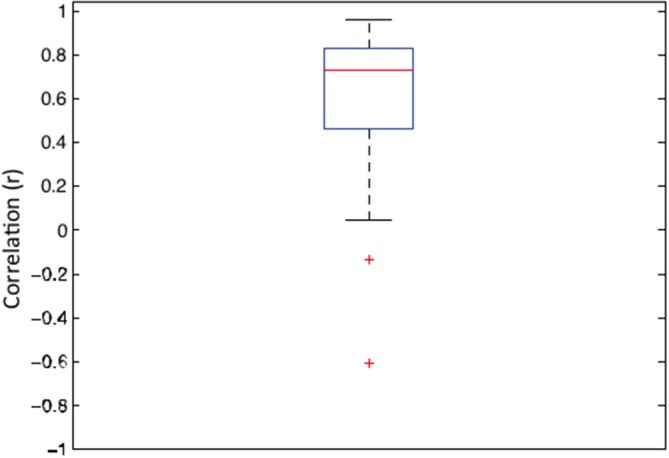
**Box and whisker plot of correlation between detection sensitivity and visual preference for sine-wave gratings for 29 observers**. In this plot, the 25–75 percent quartiles of the distribution are drawn using a box. The median is shown with a horizontal line inside the box. Maximum “whisker” length corresponds to 1.5 *Interquartile Range (25–75). This value corresponds approximately to ± 2.7 SD and 99.3% coverage. The points outside of this range are considered outliers and are denoted by red crosses.

As in the previous experiments, the correlation between average sensitivity and average preference was high (*r* = 0.876, *p* < 0.012). Similarly, the mean of individual correlations between sensitivity and preference was also high (*r* = 0.570, *CI* = 0.41–0.73, *p* < 0.0001). Figure 11 shows the box and whisker plot of individual correlations between sensitivity and preference for 29 participants. The median individual correlation between detection sensitivity and visual preference was 0.730.

Overall, the association between visual sensitivity and visual preference for sine-wave gratings at both group and individual levels was greater than that observed in our first two experiments. This might be related to the greater relative simplicity of sine-wave gratings, affording less ambiguous characterization of sensitivity for the specific parametric variations in spatial structure. The simple sine wave gratings used in this study contain only one spatial frequency component unlike synthetic images defined by variations in their amplitude spectra.

## Conclusion

While there have been several suggestions linking visual preference to various aspects of visual processing, few have been directly tested as done here. In this paper we have examined the relationship between visual sensitivity and visual preference with regards to two quantitative parameters of image structure, namely, the amplitude spectrum of spatially broadband images (measured by amplitude spectrum slope), and the spatial frequency of sine-wave gratings. The effects of these two spatial parameters were investigated in two independent, methodologically identical, and directly comparable, sets of experiments. The experimental procedures in both cases involved the measurement of visual sensitivity and visual preference in the same observers using standard tasks (contrast detection task and forced-choice paired comparison, respectively). Comparative analyses of the results from these tasks were used to discern the possible relationship between visual preference and visual sensitivity.

With respect to our investigation of variations in amplitude spectrum slope, we considered the multi-scaled structure in grayscale synthetic noise images. The results from the visual sensitivity and visual preference tasks performed on these images were consistent with the existing literature. That is, our findings that both absolute and discriminative visual sensitivity was greatest for 1/f patterns with intermediate exponent values of their amplitude spectra confirm previous findings (Knill et al., 1990; Hansen and Hess, 2006). Also, our data suggest that maximal absolute and discriminative sensitivity fall within the generally acknowledged range characteristic of natural scenes (Tolhurst et al., 1992). The visual preference also peaked for the intermediate 1/f patterns with intermediate exponent values, generally consistent with the previous observations (Spehar et al., 2003; Juricevic et al., 2010; Menzel et al., 2015). Comparative analysis between visual sensitivity and visual preference was in general supportive of our starting hypothesis, with both sensitivity and visual preference functions exhibiting inverse U-shaped patterns with peaks within similar ranges of amplitude spectra characteristics (though not always identical).

Initially, we focused on parametric variations in the amplitude spectra of two-dimensional grayscale images. However, it is possible that the same relationship could apply to even more diverse patterns and scenes that feature scale-specific spatial variations. Indeed, the purpose of our experiments, measuring visual preference and visual sensitivity in sine-wave gratings varying in spatial frequency, was to test our hypothesis that the relationship between preference and sensitivity could extend to other types of variations in image structure, beyond amplitude spectrum slope. Results from both the visual sensitivity and visual preference measurements with these patterns were consistent with the general characteristics of the contrast sensitivity function. Regarding the comparative analysis, the agreement was even closer than that found in the first set of experiments, with psychophysical functions closely matched and high individual correlations found.

Taken together, we believe that our findings support the notion that visual mechanisms involved in the processing of visual information at different spatial scales mediate visual preference. Also we believe that, the findings reported here are consistent with the view that aesthetics is not a product of the image properties *per se* (Birkhoff, 1933) but that aesthetic response is associated with the perceptual processing of such image properties. While some previous studies have already suggested the relationship between aesthetic preference and visual processing of symmetry (Bertamini et al., 2013), figure-ground contrast (Reber and Schwarz, 2001), and visual priming (Bar and Biederman, 1998), our contribution is unique in identifying an association between visual preference and the earliest stages of visual processing such as detection and discrimination sensitivity. Indeed, both the theoretically postulated and the empirically demonstrated effects of perceptual fluency are mostly concerned with the observers’ previous experience with the image (mere exposure, familiarity) or are critically dependent on a very specific and restricted set of situational characteristics associated with the viewing and/or rating of experimental images (Reber et al., 2004). Our approach extends the perceptual fluency account of aesthetic appeal to include the very early perceptual mechanisms involved with the primary coding and initial processing of images. As such it is most compatible with the “interactionist” view that considers visual preference a result of both the objective properties of external stimuli (fractal statistics) and the observer’s internal characteristics (Reber and Schwarz, 2001; Zaidel, 2015).

With respect to the question of the prevalence of natural fractal statistics in artworks, our findings also suggest a hybrid of the perceptibility and affective hypotheses formulated by Graham and Field (2008) in that efficient perceptual processing and aesthetic appeal are seemingly tied together. The same hybrid of efficient processing and associated aesthetic appeal extends to complementary observations of the considerable aesthetic appeal of natural and synthetic fractal patterns.

In summary, aesthetic experiences are undoubtedly complex and diverse in magnitude, and we are by no means suggesting that they can be explained in their entirety in relation to low-level sensory processing. We show that aesthetic judgments of spatial patterns can be directly linked to our visual sensitivity to precisely defined spatial aspects of image structure, and we believe that revealing the role of early perceptual processing in aesthetic experiences provides valuable insight and contribution to our understanding of this ageless, vital, and enriching experience. Further investigations will be useful in providing a broader and richer understanding of the relationships between early visual processing and visual preferences and aesthetic experiences.

## Conflict of Interest Statement

The authors declare that the research was conducted in the absence of any commercial or financial relationships that could be construed as a potential conflict of interest.
